# Medical College of Ohio

**Published:** 1847-10

**Authors:** 


23. *Medical College of Ohio.*—Dr. L. M. Lawson has been appointed professor of Materia Medica, Therapeutics and General Pathology in this school, and has accepted the appointment. He resigns his professorship in Transylvania University.—*Ibid.*

